# Parkinson Patients’ Initial Trust in Avatars: Theory and Evidence

**DOI:** 10.1371/journal.pone.0165998

**Published:** 2016-11-07

**Authors:** Andrija Javor, Gerhard Ransmayr, Walter Struhal, René Riedl

**Affiliations:** 1 Department of Neurology II, Kepler University Clinic, Johannes Kepler University, Linz, Austria; 2 School of Management, University of Applied Sciences Upper Austria, Steyr, Austria; 3 Department of Business Informatics–Information Engineering, Johannes Kepler University, Linz, Austria; Universita degli Studi di Perugia, ITALY

## Abstract

Parkinson’s disease (PD) is a neurodegenerative disease that affects the motor system and cognitive and behavioral functions. Due to these impairments, PD patients also have problems in using the computer. However, using computers and the Internet could help these patients to overcome social isolation and enhance information search. Specifically, avatars (defined as virtual representations of humans) are increasingly used in online environments to enhance human-computer interaction by simulating face-to-face interaction. Our laboratory experiment investigated how PD patients behave in a trust game played with human and avatar counterparts, and we compared this behavior to the behavior of age, income, education and gender matched healthy controls. The results of our study show that PD patients trust avatar faces significantly more than human faces. Moreover, there was no significant difference between initial trust of PD patients and healthy controls in avatar faces, while PD patients trusted human faces significantly less than healthy controls. Our data suggests that PD patients’ interaction with avatars may constitute an effective way of communication in situations in which trust is required (e.g., a physician recommends intake of medication). We discuss the implications of these results for several areas of human-computer interaction and neurological research.

## Introduction

Parkinson’s disease (PD) is a common, chronic progressive disease with a median annual incidence of 14 per 100.000 increasing to 160 per 100.000 in the age group over 65 [1]. Typical symptoms include bradykinesia, resting tremor, rigidity, and impaired postural reflexes [2]. In addition to these ‘cardinal motor symptoms’, non-motor symptoms occur, even in the early stages of the disease [3]. Cognitive and behavioral disturbances include deficits in the following domains: attention, memory, visuospatial functions and decision-making [4], as well as impairment of face recognition (e.g. [5]), risk-taking (e.g. [6]), trust [7], and ability to explain and predict other people’s behavior (referred to as Theory-of-Mind or mentalizing, e.g., [8, 9]). The underlying pathology is a dopaminergic cell loss in the substantia nigra pars compacta. In addition, neuronal loss is reported in the following brain areas: the dorsal motor nucleus (DMN) of the vagus, the olfactory bulb, the caudal raphe nuclei and the locus ceruleus [10]. Also, PD leads to the occurrence of Lewy bodies and neurochemical alterations in the brainstem, limbic system, diencephalic and basal brain structures, and in advanced stages, the neocortex [10]. Importantly, all mentioned deficits and impairments may negatively affect the social life of PD patients. In fact, cognitive impairment has been shown to predict quality of life in PD patients and often troubles patients more than their motor symptoms [11].

Patients suffering from all kinds of illnesses are frequently using information technology (IT) for private and disease specific information search and communication [12]. However, interaction with computers and the Internet may provoke a perception of complexity and uncertainty [13]. Evidence shows that nearly 80% of computer users with PD, report significant, severe, or highly severe difficulties using a computer [14]. Underlying factors identified in this study are inertia, muscle stiffness, tremor, and issues related to the use of input devices, among other aspects related to ergonomics. To overcome impairments, the design of human-computer interfaces for disease populations has become a major topic in human-computer interaction (HCI) research [15]. While the effects of motor symptoms on PD patients’ interaction with computers has received considerable attention in the HCI domain (e.g. [16]), behavioral symptoms and their effects have largely been neglected. However, there is growing evidence that computer and Internet use may help to provide essential information, reduce loneliness, and increase social contact among older adults in assisted and independent living communities [17]. This finding is of paramount importance for PD patients, because they frequently suffer from social isolation [18].

### Avatars and social behavior in HCI

In the HCI domain, avatars are defined as user-created digital representations symbolizing the user’s presence in a virtual environment [19]. In addition, artificial faces and characters might be part of a computer interface. In this case, characters are created by systems engineers and controlled by computer algorithms (and not by a human user). Characters controlled by algorithms are referred to as agents [19]. Examples for agents are virtual salespersons in an online shop or digital tutors in e-learning environments. To avoid complex sentence structures, we only use the term avatar in the present paper. However, we note that in this paper we refer to both avatars and agents.

Social information processing theory [20] postulates that users engage in interpersonal processes online in order to attain interpersonal goals. A major goal of communicators is the reduction of uncertainty about others [21]. Visual appearance plays a significant role in the formation of first impressions of strangers, and this visual and nonverbal information is processed to form a first impression within seconds [22]. Thus, provision of visual information may significantly reduce uncertainty in social interaction. Importantly, first impressions are frequently very resistant to change [23]. It follows that first impressions significantly affect future behavior of communicators in the social interaction process. In online environments, visual information is often provided based on static information (e.g., by showing a communicator’s face or body [24]). Importantly, avatars are able to induce trust and reduce uncertainty despite being presented statically, or as an animation [25, 26].

There is vast evidence on how healthy humans interact with avatars. Several studies based on healthy subjects have demonstrated that avatars are often perceived as social agents, and thus avatars may socially influence humans. Hence, humans interacting with avatars have an experience of being with another person [27] and are willing to disclose information that is considered highly personal, even when avatars are low in visual realism [28]. Moreover, recent evidence indicates that avatars are trusted to a similar extent as humans [29]. Thus, avatars help to overcome perceptions of uncertainty in computerized environments by imitating face-to-face interaction [30].

In addition to research on healthy subjects, there have been first studies to investigate how patients suffering from psychiatric diseases, such as schizophrenia (e.g. [31]) and autism (e.g. [32]), interact with avatars. However, to the best of our knowledge, neither does a peer-reviewed scientific journal article focus on neurological patients’ interaction with avatars, nor does peer-reviewed research at the nexus of avatars and PD exist. This finding is surprising, because avatars are used in (i) computer systems that are specifically designed to evaluate PD patients [33], (ii) assistive technologies [34], and (iii) neurorehabilitation systems for PD patients [35].

To close this significant research gap, we investigate the trust behavior of PD patients during their interaction with avatar and human faces in a trust game context. In this game, originally developed in behavioral economics [36], an investor (also referred to as trustor) receives an initial monetary endowment (e.g. €10). The investor decides how much of this initial endowment he or she would like to send to another player in the game, the trustee. This amount is then multiplied by the experimenter (e.g. €10 x 6 = €60). In the next move, the trustee decides whether to send money back to the investor (and, if so, how much). The amount of the investor’s transfer is a behavioral measure of initial trust [36–38].

In the next section, we give an overview of (1) the pathoanatomical basis of major behavioral symptoms in PD and (2) the vast literature on the neurobiology of trust in both interpersonal and human-avatar interaction.

### Behavioral symptoms in PD

Behavioral and cognitive neurology, a field that studies behavioral symptoms and cognitive functions in patients suffering from neurological disorders, has identified several impairments in PD. Cognitive symptoms of PD primarily affect executive functions [39]. It is still a matter of research why some PD patients develop cognitive impairments in addition to the cardinal motor symptoms in early stages of the disease, while others do not, or only at a very late stage of the disease. The reason for this observation might be that motor symptoms and cognitive deficits have distinct underlying anatomical bases [40]. In the following, we review the PD literature on pathological changes in brain regions relevant to trust, and we relate these regions to cognitive and behavioral impairments in PD.

First, PD pathologically affects the limbic system, particularly the amygdala and the thalamus (e.g. [41, 42]). A network of brain structures including the amygdala underlies the ability of face recognition [43]. This explains why PD patients have been shown to have problems in decoding facial expressions and hence perform significantly worse in recognizing sadness, anger, and disgust in human faces when compared to healthy controls (e.g. [44]).

Second, in PD, the loss of dopaminergic neurons in the substantia nigra leads to pathological functioning of striatal structures [45] and to impairments in the meso-cortico-limbic dopaminergic system, including the ventral tegmental area, ventral striatum, and medial orbitofrontal cortex [46]. Moreover, a network of brain structures that involves multiple cortical and subcortical regions (prefrontal, parietal, limbic) constitutes the neural basis of decision-making under risk or uncertainty [47]. This network overlaps in critical parts with the reward system (striatum and frontal cortical regions, e.g. [48]), and therefore explains altered risk processing in PD patients. Drug naïve patients are typically risk averse, while dopaminergic treatment makes these patients more prone to risk-seeking behavior (for a review, see [6]).

Imaging data also suggests that frontal cortical regions are affected by PD (e.g. [49]), and frontal lobe functions are disturbed in PD in about 30% of the patients [3]. Impairments in mentalizing (Theory-of-Mind) have also been reported for PD patients (for a review, see [50]), and these disturbances are mainly caused by frontal cortex dysfunction, because the frontal cortex is considered a key region for the neural implementation of mentalizing (e.g. [8]). A recent review of 13 studies focusing on mentalizing in PD concludes that (i) deficits are common and (ii) those deficits probably play an important role in daily living and affects quality of life in these patients [9].

To sum up, we have briefly reviewed evidence on the pathological changes of the limbic system, the basal ganglia, and the frontal cortex in PD. Importantly, human trust relies on all of these brain regions (for a review, see [51]), and PD patients have recently been shown to have lower trust in other humans than healthy controls [7].

Next, we provide a short overview of the literature on the neurobiology of trust.

### The neurobiology of trust

Trust is a fundamental prerequisite for human relationships, both in private and public life, and thus is essential for the functioning of society in general [52]. It is important to differentiate trust behavior from its antecedents (beliefs about trust, behavioral intentions, and trust disposition) as these concepts are frequently mixed up in the literature [53]. Following the behavioral intentions concept, Rousseau et al. [54] defined trust as a psychological state comprising the intention to accept vulnerability based on positive expectations about the actions of another party (the trustee). Formally, trust is therefore the subjective assessment of the probability that someone is trustworthy [55]. Trust *behavior* has been defined as trusting acts influenced by beliefs about another person’s trustworthiness and risk preference [56]. Initial trust is trust a person has in a stranger before having any interaction experience with that person. This kind of trust plays an important role in online environments, as users often have little common history or may not share the same cultural background [57]. Knowledge-based trust, in contrast, is trust that emerges through engagement with the other person. While initial trust strongly depends on the characteristics of another person (e.g. face), knowledge-based trust primarily develops from another person’s reciprocal behavior. One person, the trustor, is acting in a way that makes him vulnerable to the actions of another person, the trustee, in the sense that trust might not be reciprocated. Due to a social preference referred to as”betrayal aversion“, humans generally take risks less willingly when the cause of uncertainty is another person, and this fact differentiates trust from non-social risk-taking (e.g. [58, 59]).

Trust has become a major research topic in various scientific disciplines, including economics, neuroscience, medicine and information systems research (e.g. [58, 60, 61]). Neuroscience has used several methodological approaches (e.g. functional brain imaging) to detect the neural correlates of trust. In the following, we summarize key findings from an anatomical point of view and, in order to enhance understandability from a non-neuroscience perspective, group several smaller brain areas into three main regions that were found to be significantly activated in trust situations, namely the limbic system, basal ganglia, and the frontal cortex (for more comprehensive reviews, see [51, 58, 62]).

The limbic system is a term used for several brain regions, including the amygdala and the hippocampus that are strongly related to emotions and memory [63]. The amygdala is active during the assessment of the trustworthiness of human faces (e.g. [64–66]), a fact that holds particularly true for the assessment of untrustworthy faces [43, 66]. A study based on the trust game found that patients with unilateral amygdala damage showed more trust than healthy controls [67]. This finding suggests that the amygdala plays a crucial role in trust behavior between humans.

The basal ganglia are a subset of subcortical nuclei that are interconnected with several cortical areas (especially the frontal cortex), as well as the thalamus and the brainstem. The basal ganglia have important functions for the motor system, but also for learning, cognition, and behavior (e.g. [68]). One part of the basal ganglia is the striatum, which is active in trust situations because the striatum is associated with reward processing and reward anticipation (e.g. [64–69, 70]). The caudate nucleus, which is also part of the basal ganglia, has also been shown to be active in trust game studies [65].

Theory-of-mind brain regions are needed to predict another person’s trustworthiness based on anticipation of intentions and future behavior. The outcome of this process is known as ‘calculative trust’ [71]. Theory-of-mind regions that have been shown to be active in trust behavior are the paracingulate cortex [72] and the medial prefrontal cortex [73]; we group both regions together and refer to them as frontal cortical regions in our paper. These regions seem to have distinct functions in trust behavior, as the paracingulate cortex has a role in building a trust relationship by inferring another person’s intentions to predict subsequent behavior, and the medial prefrontal cortex has implications for decisions and choices based on calculative expectations of what others will do [74].

### Hypotheses

**Fig 1:**
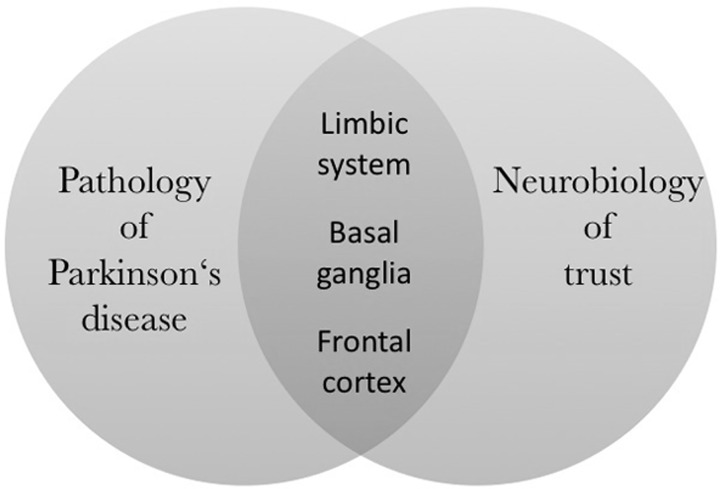
Brain regions related to trust and affected by PD.

We provided an overview of the most important aspects of the biological foundations of social behavior deficits in PD and the neurobiology of trust. It becomes apparent that there is a significant overlap between pathoanatomical changes in PD and the anatomical basis of trust. We visualize this overlap in Fig 1.

**Fig 2:**
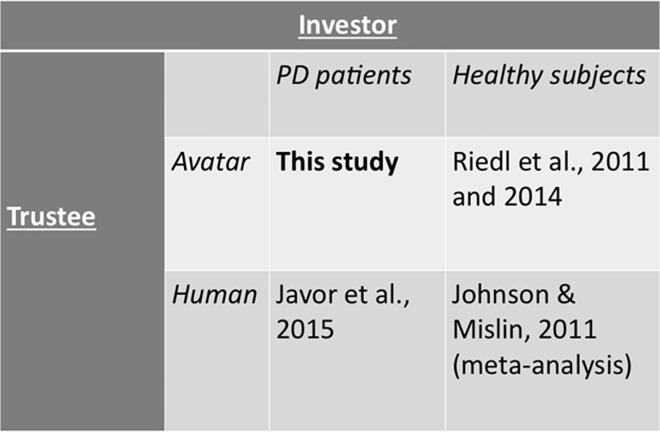
Visualization of current knowledge of trust research and the contribution of the present study.

In order to develop a theoretical basis for our hypotheses that describe how PD patients are expected to behave in a trust situation with avatars, we summarize evidence on PD patients’ trust in human faces and healthy controls’ trust in avatars and human faces in Fig 2. Based on this conceptualization, we identify two significant gaps in literature. In the following, we develop hypotheses for these two gaps. Based on our preceding discussion of neuroscience research, we argue as follows:

1. Trust is mainly represented in three brain regions, namely the limbic system, the basal ganglia, and frontal cortical regions; PD affects all these regions. It has been shown that PD patients have lower initial trust in simulated face-to-face interactions compared to healthy controls [7].
2. There is evidence that mentalizing brain regions are more active during the evaluation of the trustworthiness of human faces if compared to avatar faces [29]. Furthermore, avatar faces elicit less activation in the limbic system if compared to human faces [26].
3. One could argue that even though PD patients show lower initial trust in simulated face-to-face interactions than healthy controls [7], these patients have less or even no trust deficit when interacting with avatars, because brain regions that are regulating trust behavior (e.g. medial frontal cortex and the amygdala) are less active in the interaction with avatars compared to interaction with humans [26, 29]. Therefore, impairments in these regions caused by PD should have less impact on trust behavior towards avatars.

Following this line of argumentation, we formulate the following hypotheses that we tested in a laboratory experiment:

H1: PD patients show higher initial trust in avatar faces than in human faces.
H2: PD patients show similar initial trust in avatar faces if compared to healthy controls.

## Methods

This study was approved by the ethics committee of Upper Austria. Informed written consent was obtained from all participants prior to the experiment.

### Participants

Forty right-handed, Caucasian participants were recruited for the study. Twenty of the subjects were patients diagnosed with PD according to UK PDS Brain Bank criteria [75], an instrument with a diagnostic accuracy of 90% [76]. The patients were recruited at an outpatient clinic specialized in movement disorders (mean age 72.35 years, SD 9.16, equally gendered). The same patient population was studied before and more clinically relevant variables are available elsewhere (see [7]). This study is part of an extended project to explore trust in PD. All patients were clinically stable under treatment prior to the experiment. Healthy controls had a mean age of 68.4 years (SD 10, also equally gendered), no history of neurological disease, and a neurological status with no pathological findings. There was no significant difference between PD patients and healthy controls with respect to: age (two-sided unpaired t-test; t = 1.3, p = 0.20), income (classified in 6 categories; median patients: 4; median controls: 3; Mann-Whitney-U-test; z = -0.86; p = 0.39), education (two-sided unpaired t-test; mean education patients: 10.9 years, SD 4.02; mean education controls: 11.3 years, SD 5.11; t = -0.28; p = 0.79), and religion (all participants were Roman-Catholic); note that these factors might affect trust behavior [77].

We screened for psychiatric co-morbidities like dementia in all patients and controls, as well as for impulsive-compulsive disorders, and/or apathy in all patients and excluded those meeting pathological criteria. As screening tools we used the complete version of the Patient Health Questionnaire (PHQ-D, [78]), the Questionnaire for Impulsive-Compulsive Disorders (QUIP, [79]), the Mini Mental State Examination (MMSE, [80]), and the Lille Apathy Rating Scale (LARS, [81]). To quantify the severity of symptoms in PD we also used the Unified Parkinson's Disease Rating Scale (UPDRS, [82, 83]), a scale commonly used in clinical practice. The mean UPDRS-III score of patients under treatment was 19.85 (SD 10.59). For a summary of the characteristics of our study population see Table 1.

**Table 1:** Characteristics of the patient and control groups.

*Characteristics*	*PD patients*	*Healthy controls*
Number of subjects (n)	20	20
Female, n (%)	10 (50)	10 (50)
Age (years)	72.35±9.16	68.4±10
Education (years)	10.9±4.03	11.3±5.11
MMSE	28.4±1.6	28.95±0.99

Abbreviations: MMSE: Mini Mental State Examination. Except where indicated, values are means plus/minus standard deviation.

### Experimental Procedure

We designed a behavioral experiment to test our hypotheses. When subjects entered the laboratory for the experiment, they received written instructions explaining the rules and payoff structure for the experiment (the instructions are available from the first author on request). The experimenter subsequently checked whether the subjects understood the instructions by going through several hypothetical examples. All subjects correctly answered the control questions.

We measured trust using a version of the original trust game [36]. The trust game was developed to measure trust as actual behavior of players in an economic exchange game. In this game, one player (the trustor), has an initial endowment of x monetary units. First, the trustor decides whether to keep his or her endowment, which ends the game, or to send (a part of) it to a second player (the trustee). The trustee observes the trustor’s action and, if money was sent, decides whether to keep the amount or share (some of) it with the trustor. The experimenter multiplies the trustor’s transfer, so that both players are better off collectively if the trustor transfers money and the trustee sends back a sufficient amount. In the trust game, the amount sent by the trustor is used as a behavioral measure for trust [36–38].

**Fig 3:**
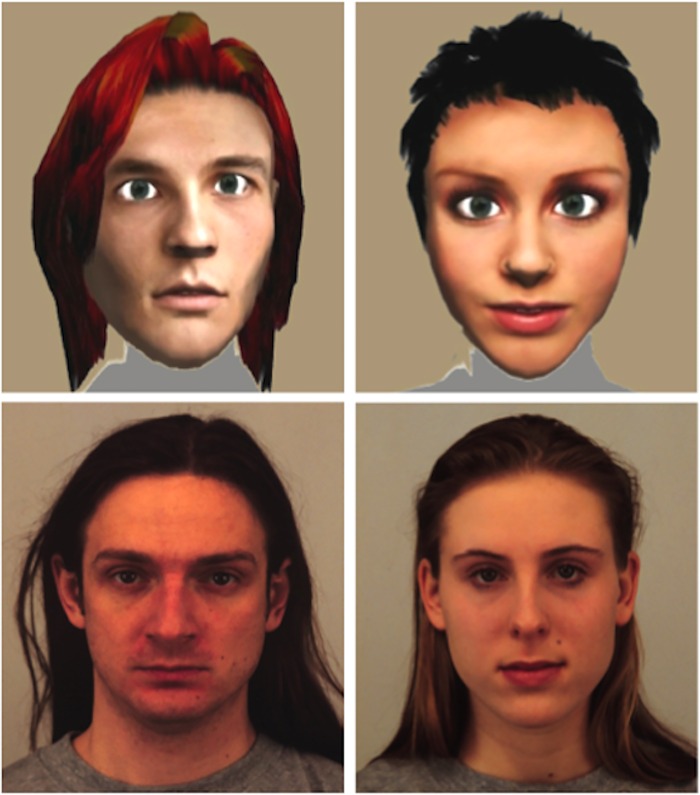
Examples of stimulus material used in this study.

Our version of the game focused on the trustor’s behavior, whose role was played by the participants on a computer. The participants were told that they would be playing against human beings presented in the form of face photographs or avatar faces, but actually played against an automatic and randomized computer-generated strategy that simulated a trustee’s behavior (see Fig 3 for examples of human faces and avatar faces which we used as stimulus material).

**Fig 4:**
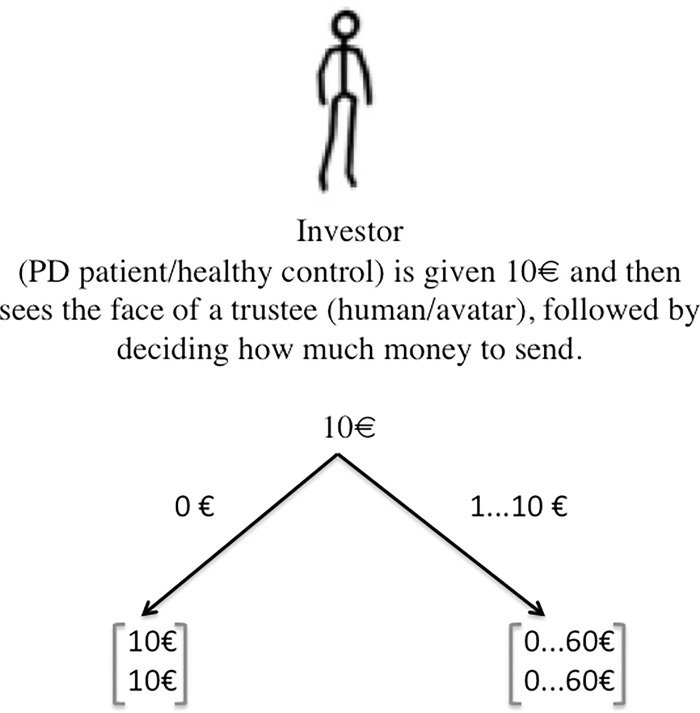
Illustration of our version of the trust game. Notes: The upper value in the square brackets indicates the investor’s payoff (any amount between €0 and 60), the lower value the trustee’s payoff depending on the investor’s first move (investment of €0 or any amount between €1 and 10). When the investor is not sending any money to the trustee (€0) the payoff is €10 for both players. In any other case, the investor’s payoff is dependent on the trustee’s willingness to send some money back. Whereas the investor’s payoff determined the overall gain of the participants in our study, the trustee’s payoff was not paid out, as this role was not played by participants, but was part of the computerized experiment (see Experimental procedure).

The initial endowment of the participants was €10, and they were told that they would be playing with real money and that the recompense for participating in the experiment would reflect their gain in the game. The participants could decide to send any amount to the trustee but had to use whole numbers between €0 and €10. This amount sent by the participants was used to operationalize initial trust. The trustee was illustrated with 16 human face images (taken from an established face database [84, 85]) and 16 avatar faces (taken from the stimulus material from published research [29, 86]). The selection of the human and avatar stimuli ensured the same face trustworthiness and gender in both groups. None of the human faces or avatar faces were known or previously seen by the participants. It follows that participants had no interaction experience with the stimulus material. In total, each subject made 32 decisions in the experiment, and the order of face presentation was completely random. It follows that no participant interacted with the same trustee twice. We used the monetary amount sent by each participant as a measure of trust behavior. Fig 4 illustrates our version of the trust game. We included a gambling task (Game-of-Dice task, for a detailed description of the game see [4]) in our experimental design, because non-social risky decision-making is considered a standard control condition in trust research (e.g. [87]).

### Statistical Analysis

We used SPSS^®^ (Version 20) to perform statistical analyses. Descriptive statistics were run to illustrate the demographic characteristics of the sample and to describe disease specific information about the patient group (e.g. UPDRS-II scores). A Kolmogorov-Smirnov test revealed that game behavior of both experimental groups conformed to normality and a Levene’s test of equality assured that there was homogeneity of variances between groups. Unpaired sample two-sided t-tests were performed to compare game behavior between groups and paired sample two-sided t-tests to compare within-group behavior between the human face and avatar face conditions.

## Results

**Fig 5:**
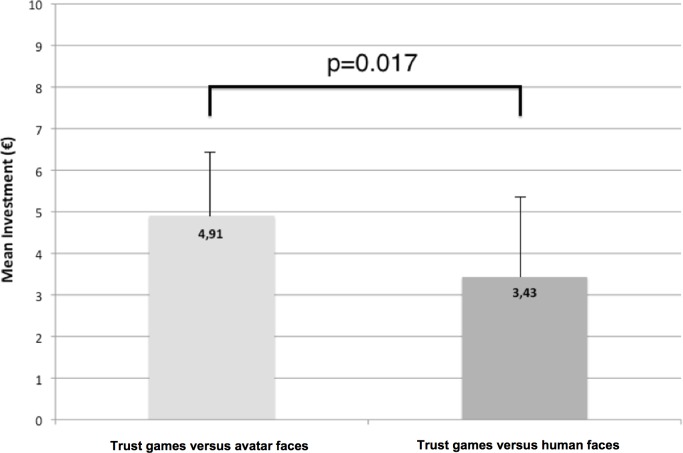
Results of overall mean investments in games versus avatar faces (left) and human faces (right) of the PD patients group.

H1 states that PD patients show higher initial trust in avatar faces than in human faces. H1 is supported by our data. PD patients invested significantly more in trust games played against avatar faces versus games played against human faces (mean avatar faces: €4.91, SD 1.55; mean human faces: €3.43, SD 2.00; two-sided paired sample t-test; t = 2.62; p = 0.017, see Fig 5). In contrast, healthy controls’ mean investment amounts did not differ significantly in trust games against avatar faces versus human faces (mean avatars: €4.94, SD 1.55; mean human faces: €5.53, SD 1.56; two-sided paired sample t-test; t = -1.53; p = 0.141).

**Fig 6:**
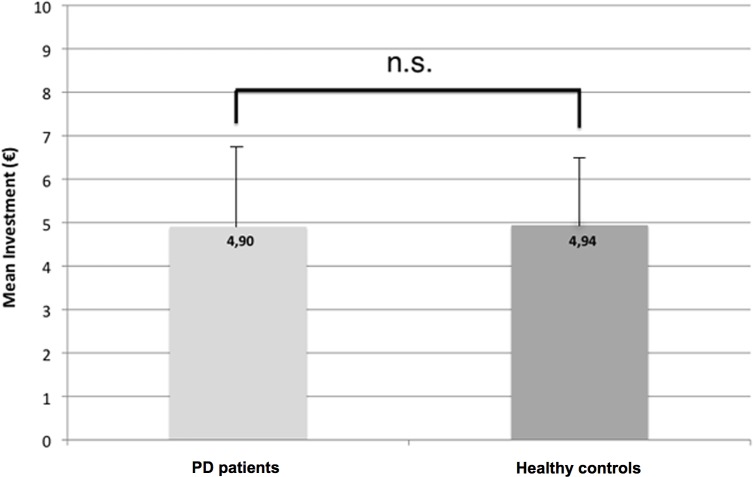
Results of overall mean investments in games versus avatar faces of PD patients (left) and healthy controls (right).

H2 states that PD patients show similar initial trust in avatar faces if compared to healthy controls. H2 is also supported by our data. There was no significant difference in the mean investment amount between the PD patients group and the control group in trust games played against avatar faces (mean patients: €4.91, SD 1.81; mean controls: €4.94, SD 1.55; two-sided unpaired t-test; t = -0.07; p = 0.949; see Fig 6). Further analyses to determine the effect size of this condition revealed a Cohen’s d = 0.02 and an effect size r = 0.01.

PD patients, if compared to healthy controls, invested significantly less in games against human faces (mean PD patients: €3.43, SD 1.996; mean controls: €5.53, SD 1.56; two-sided unpaired t-test, t = -3.70; p = 0.001). The Game-of-Dice Task showed a significant difference in risk behavior between the patient group and the control group, with a larger number of high-risk choices (out of 18 choices) in the PD patient group (mean PD patients: 10.2, SD 3.928; mean controls: 7.05, SD 3.620; two-sided unpaired t-test; t = 2.637, p = 0.012).

## Discussion

The results of our study confirm H1 that PD patients have significantly higher initial trust towards avatar faces when compared with human faces. Our data further support H2, which states that there is no significant difference between initial trust levels towards avatar faces between PD patients and healthy controls. Although it cannot be ruled out that a larger sample size could lead to a significant difference in this condition, we performed additional statistical analyses (Cohen’s d), which indicates that there is indeed a negligible difference between mean investments of PD patients and healthy controls (see Fig 5). This is an intriguing result, because PD patients are known to invest less in a trust game, if compared to healthy controls, when playing against human faces [7]. Our control condition (Game-of-Dice task) confirms that lower trust in human faces is not a consequence of higher risk aversion in PD. Because of our study design (emotionless human faces and avatar faces, as well as no difference in colors used in the stimulus material) we can rule out effects of emotion recognition deficits (e.g. [88]) or color discrimination problems [89], known to affect PD patients, on our data.

Even though the results of our behavioral study do not make it possible to determine the underlying neurological mechanisms of the observed trust differences, our results have important implications for HCI in general and for specific HCI technologies used in medicine. Because evidence shows that the belief of interacting with either an avatar (user-controlled virtual character) or an agent (machine-controlled virtual character) does not result in significant differences with regard to the evaluation of the virtual character nor in different behavioral reactions [90], our discussion of implications is relevant for interaction of PD patients with both avatars and agents.

The findings of the present study are relevant to both computer scientists and programmers with a focus on HCI research, as well as medical scientists and physicians. Therefore, we discuss our results from a multidisciplinary perspective. We start the following discussion with HCI, where we group the implications in e-commerce, e-learning, and social media. A reflection on our results from the perspective of computer use in medicine follows, grouped into diagnosis and therapy in PD and patient-physician communication. Following the implications sections, we describe limitations of our study and close the paper with final remarks.

### Implications for HCI

Avatars are used in several domains of HCI, such as e-commerce, e-learning, and social media.

In e-commerce, avatars can lead to a higher satisfaction with online retailers [91], because they can substitute a real-life salesperson (in the sense of an automated online assistant) and thereby reduce information overload [92], provide recommendations on suitable products [93], and enhance consumer decision-making (e.g. [94]). Furthermore, avatars have a significant effect on how consumer reviews are perceived by consumers [95]. This is important, because consumer reviews have a major impact on purchase decisions (e.g. [96]). Our data implies that online shops frequently used by PD patients could increase trust by using avatars as virtual sales consultants. It has been argued by Javor et al. [60] that all research involving patients or disabled persons and possibly affecting marketing should be evaluated from an ethical point of view, as disease specific weaknesses of these populations can be subject to target marketing. Our study might raise concerns whether PD patients can be targeted by online marketing measures using avatars. Importantly, the results of our study show that avatars are trusted to a similar extent by PD patients and healthy controls. Hence, the interaction of PD patients and avatars in a trust-related context such as online shopping does not seem to be affected by the disease process and therefore, in our understanding, the use of avatar salespersons in the interaction with PD patients is ethically uncritical.

E-learning is another application domain of avatars. In recent years, this domain has grown considerably, and the market for learning management systems has also exceeded growth predictions, currently worth around $2.55 billion worldwide according to the E-Learning Market Trends & Forecast 2014–2016. The use of virtual environments and avatars in e-learning systems play a key role in creating a sense of presence for users in the shared environment [97]. Grujic et al. [98] argue for the use of a virtual tutor in an electronic educational system. The tutor’s major aim is not necessarily to teach students, but to emotionally respond to their actions with gestures and facial expressions. In a randomized controlled trial to evaluate the effectiveness of a patient education and health promotion program in the treatment of Parkinson's disease, the intervention group had significantly increased exercise, decreased “time off” (i.e., time periods of increasing symptoms mostly due to medication effects) and percentage of time off, reduced side effects, and decreased summary Parkinson's scores by approximately 10% [99]. There have been efforts to use e-learning for patient education in neurological patient populations, but these have not shown comparable results to face-to-face education (e.g. [100]). Given the positive effect of trust in the teacher on student performance [101], our data might help to improve patient education through e-learning by the use of avatars as tutors. Research in the field of Alzheimer’s disease has already shown that patients suffering from neurological diseases might benefit from interaction with avatars [102]. Our results suggest that this finding could also hold true in the population of PD patients.

Avatars have also found their way into social media environments such as Facebook [103]. A recent review focusing on studies about the use of social media in patients and caregivers identified discussion forums as being highly popular (66.6%), and social networking sites (14.8%) and blogs/microblogs (14.1%) were the next most prevalent applications [104]. Several researchers highlight the importance of trust in social media environments (e.g. [105]). Dubois et al. [106] state that “[w]ith so much user interaction and content created, the question of whom and what to trust has become an increasingly important challenge on the web. A user is likely to encounter dozens if not hundreds of pieces of user-generated content each day, and some of it will need to be evaluated for trustworthiness. Trust information can help a user make decisions, sort and filter information, receive recommendations, and develop a context within a community with respect to whom to trust and why”(p.1).

A review of public health messages through social media indicates that more and more consumers turn to the internet for health related information and health organizations have therefore begun to turn to social media as a tool for connecting with the public, but only a very limited number of studies have analyzed the efficacy of social media in this context so far. Preliminary reports point to considerable outreach of social media applications and have the potential for engaging specific target audiences [107]. We argue for the use of avatars in public health social media messages aimed at PD patients in order to increase trust towards these contents. We could not identify a scientific study on the use of social media by PD patients. Hence, we make a call for research in this domain. It has already been discussed that avatars could be used to facilitate access to technologies for the elderly [108]. Our data support the idea of integrating avatars in the design of social media platforms to increase PD patients’ trust, thereby increasing the inclusion of these patients in online social interaction.

### Implications for the use of computers in medicine

In the medical field, several applications of avatars have emerged (e.g., diagnosis and therapy of neurological disorders and patient-physician communication).

Avatars have been used in the diagnosis and therapy of neurological disorders for almost 20 years [109]. An important part of therapy in PD is physical exercise that has been shown to positively affect physical functioning, health-related quality of life, strength, balance, and gait speed of PD patients [110]. Problems frequently occurring in intensive daily training are often motivational. Virtual environments have been used frequently in rehabilitation of PD in recent years to overcome these problems, because they contribute to motivating patients to exercise more [111]. In PD, two aspects of rehabilitation are of major concern. First, the above mentioned cardinal motor symptoms of the disease are being targeted. In this context, virtual environments often use avatars to represent the PD patient (e.g. [112]). In the light of evidence from cyberpsychology that players of online role-playing games sometimes identify themselves more strongly with the avatar compared to the real self (e.g. [113]), PD patient self-representation with an avatar seems to be an effective way to enhance rehabilitation. Second, behavioral and cognitive rehabilitation of non-motor symptoms (e.g. social interaction for behavioral symptoms or playing “Sudoku” for cognitive symptoms) [114] plays an important role, and avatars are often used in this context (e.g. [115]). Previous literature argues for the use of avatars in the treatment of mentalizing deficits of patients suffering from autism [116], and we argue that patients with PD might also benefit from such a training. Further, given the importance of trust in rehabilitation [117], our data supports the use of avatars in both motor and cognitive rehabilitation of PD patients.

With respect to avatars in patient-physician communication, a recent study advocates telemedicine in the care of PD patients and is arguing that”[t]ravel distance, growing disability, and uneven distribution of doctors limit access to care for most Parkinson's disease (PD) patients worldwide. Telemedicine, the use of telecommunications technology to deliver care at a distance, can help overcome these barriers”[118]. It has previously been argued that avatars could be used in patient communication to promote behavioral change, such as life style modification [119]. Our data show that avatar faces are trusted significantly more than human faces by PD patients, and this level of trust is similar to that of healthy controls. It can be theorized that communication with PD patients through avatars in the context of telemedicine could promote the patient-physician relationship and therefore improve health care in PD.

### Limitations

The stimulus material consisted of unknown human faces and unknown avatar faces. It follows that results cannot be generalized to interactions with known counterparts. Furthermore, we make a call for studies replicating our investigation in order to substantiate the results of the present study.

As our study examined trust behavior of PD patients towards avatar faces (i.e., patients acted in the role of investor in our trust game), there is still missing evidence how PD patients would behave in the trustee role (i.e., the party who is given trust and subsequently has to reciprocate, or not). Thus, we make a call for future studies investigating PD patients’ reciprocation of trust. Specifically, it will be rewarding to see whether differences exist in PD patients’ interaction with both humans and avatars acting in the role of the investor. It might turn out that PD patients not only exhibit increased trust towards avatars when compared to humans; rather, it is possible that PD patients are also more trustworthy in their interactions with avatars than in their interaction with humans (i.e., they reciprocate more in the avatar condition than in the human condition in a trust game where they are given money by the investor).

Furthermore, we acknowledge that the results of the present study should be validated with a larger sample size. However, our statistically significant results based on a sample size of n = 20 in each experimental group along with a conservative level of significance (p<0.05) imply a large effect size. Additionally, the characteristics of our subjects (e.g. age, education level, all Caucasian, all Catholic) should be considered when interpreting our findings, because trust has been shown to correlate with those factors [69].

Finally, our study design using behavioral methodology does not allow for direct inferences on the exact nature or neurological basis of the differences in trust behavior of PD patients towards human faces and avatar faces. Therefore, we encourage studies using electrophysiological (e.g. electroencephalography, EEG) or functional brain imaging tools in order to shed light on the neural basis of trust behavior in PD.

## Conclusion

Our study is a first step to examine human-avatar interaction at the nexus of behavioral neurology (neurological disorders affecting behavior) and PD. Our results suggest that avatars can be used to overcome behavioral deficits caused by a neurological disease, in our case PD patients’ reduced trust in human social interaction. We hope that the present study instigates further interdisciplinary research in the domain of behavioral neurology and HCI. Advancements in this research field are urgently needed because neurological diseases and disorders are on the rise, and hence it is important to determine the positive and negative effects of IT use (e.g. in the form of avatars) for different patient populations.

## Supporting Information

S1 File File containing parts of the original data following recommendations from the local ethics committee. (XLSX)
